# A case of ultrasound-guided prenatal diagnosis of prune belly syndrome in Papua New Guinea – implications for management

**DOI:** 10.1186/1471-2431-13-70

**Published:** 2013-05-07

**Authors:** Maria Ome, Regina Wangnapi, Nancy Hamura, Alexandra J Umbers, Peter Siba, Moses Laman, John Bolnga, Sheryle Rogerson, Holger W Unger

**Affiliations:** 1 Papua New Guinea Institute of Medical Research (PNG IMR), Vector Borne Disease Unit (VBU), P.O. Box 378, Madang 511, Papua New Guinea; 2 Department of Obstetrics and Gynaecology, Modilon General Hospital, P.O. Box 2119, Madang, 511, Papua New Guinea; 3 Royal Women’s Hospital, Melbourne, VIC, 3052, Australia

**Keywords:** Congenital abnormality, Eagle-Barrett syndrome, Management, Papua New Guinea, Prenatal diagnosis, Prune belly syndrome, Ultrasound

## Abstract

**Background:**

Prune belly syndrome is a rare congenital malformation of unknown aetiology and is characterised by abnormalities of the urinary tract, a deficiency of abdominal musculature and bilateral cryptorchidism in males. We report a case of prune belly syndrome from Papua New Guinea, which was suspected on pregnancy ultrasound scan and confirmed upon delivery.

**Case presentation:**

A 26-year-old married woman, Gravida 3 Para 2, presented to antenatal clinic in Madang, Papua New Guinea, at 21^+5^ weeks’ gestation by dates. She was well with no past medical or family history of note. She gave consent to participate in a clinical trial on prevention of malaria in pregnancy and underwent repeated ultrasound examinations which revealed a live fetus with persistent megacystis and anhydramnios. Both mother and clinicians agreed on conservative management of the congenital abnormality. The mother spontaneously delivered a male fetus weighing 2010 grams at 34 weeks’ gestation with grossly abnormal genitalia including cryptorchidism, penile aplasia and an absent urethral meatus, absent abdominal muscles and hypoplastic lungs. The infant passed away two hours after delivery. This report discusses the implications of prenatal detection of severe congenital abnormalities in PNG.

**Conclusion:**

This first, formally reported, case of prune belly syndrome from a resource-limited setting in the Oceania region highlights the importance of identifying and documenting congenital abnormalities. Women undergoing antenatal ultrasound examinations must be carefully counseled on the purpose and the limitations of the scan. The increasing use of obstetric ultrasound in PNG will inevitably result in a rise in prenatal detection of congenital abnormalities. This will need to be met with adequate training, referral mechanisms and better knowledge of women’s attitudes and beliefs on birth defects and ultrasound. National medicolegal guidance regarding induced abortion and resuscitation of a fetus with severe congenital abnormalities may be required.

## Background

First described by Frölich in 1839 [1], prune belly syndrome (PBS), also known as Eagle-Barrett syndrome [2] or Obrinsky syndrome [3], is a congenital disorder affecting 2.5 to 3.8 per 100,000 live births [4,5]. It is largely but not exclusively confined to males [4,6], and is defined clinically by a combination of abnormalities of the urinary tract such as the absence or partial obstruction of the urethra and renal dysplasia, a deficiency of abdominal musculature and bilateral cryptorchidism in males [7]. Other fetal malformations which may be associated with PBS include gastrointestinal, cardiac, pulmonary and limb abnormalities [8]. Prenatal ultrasound features can include oligo- or anhydramnios, megacystis, hydronephrosis, and hyperechogenic kidneys [9]. To date, a clear genetic basis for PBS has not been established and the aetiology of the syndrome is unknown [10]. It is thought that PBS arises from a defect in the intermediate and lateral plate mesoderm development during the 6^th^ to 10^th^ week of gestation, resulting in clinical abnormalities [9,11,12].

In high-income countries, most cases of PBS are diagnosed antenatally through ultrasound, and confirmed at birth [9,13]. By contrast, in resource-limited settings such as Papua New Guinea (PNG) prenatal diagnosis of congenital abnormalities remains unreported, and documentation of congenital abnormalities observed after delivery is rare [14-17].

The use of ultrasound to complement antenatal care in PNG is relatively recent [18] and is restricted to a small number of facilities or medical research projects [19]. It depends on the presence of specialists with ultrasound training (obtained abroad), and machines are usually donated and rarely procured through governmental funds. Scans are not provided routinely as part of antenatal care in PNG; however, when available and clinically indicated, women are referred for confirmation of gestational age, diagnosis of multiple gestation, and assessment of suspected placental and amniotic fluid level abnormalities.

This is the first formal report of PBS in PNG and other resource-limited settings in the South Pacific, and was first suspected on ultrasound. It highlights challenges clinicians in PNG are facing when counseling affected parents on diagnosis and prognosis of severe congenital abnormalities detected prenatally.

## Case presentation

The mother of the patient was a 26-year-old, married, Gravida 3 Para 2 (two healthy children 5 and 3 years old, uncomplicated spontaneous vaginal deliveries at term) who presented to an urban clinic in Madang, PNG, in April 2012 for routine antenatal booking of a planned pregnancy at 21^+5^ weeks’ gestation by dates.

The mother gave informed written consent to join a randomised controlled trial that took place at the clinic at the time. The purpose of the trial was to examine the effect of intermittent preventive therapy in pregnancy (IPTp) with azithromycin and sulphadoxine-pyrimethamine (SP) on reducing the proportion of babies born with low birthweight [20,21], and women were followed up until delivery. As part of the study, participants were offered an ultrasound examination (USS) by a research clinician (Logiqbook XP, General Electric Medical Systems, Little Chalfont, UK). The main purpose of the scan was to corroborate clinical history and examination data by providing an additional, more accurate estimate of gestational age (to detect preterm births) and to screen for twin pregnancies. Scans were performed during a separate research ultrasound clinic visit within a week of enrollment into the study.

Upon presentation the mother was well and had no complaints. There was no past medical and family history of note. She was a non-smoker, had not consumed alcohol during this pregnancy, and had a normal body mass index (20.1 kg/m^2^). She reported regular fetal movements since 19 weeks’ gestation. Observations taken on the day were normal for gestation (blood pressure 88/54, heart rate: 86 beats per minute, temperature 36.5°C). On abdominal palpation the fundal height was 23 cms. A fetal heart rate of 144 beats per minute was observed, fetal lie was longitudinal and presentation was cephalic. She was mildly anaemic (haemoglobin = 9.6 g/dL) (Hemocue, Quest Diagnostics, Madison, USA) and was given dietary advice. She received IPTp with azithromycin 1g twice daily for two days and a single dose of SP of 1500/75 mg as per trial protocol.

The mother underwent three ultrasound scans (USS) in total: the first ultrasound examination was performed by a research clinician two days after initial presentation at 22^+0^ weeks, at which time the fetal abnormality was detected. The mother was withdrawn from study treatments and referred to the hospital clinical team. The research clinician conducted a further ultrasound examination at 24^+1^ weeks, which was followed by a third USS was performed at 24^+3^ weeks’ gestation at hospital.

**Figure 1:**
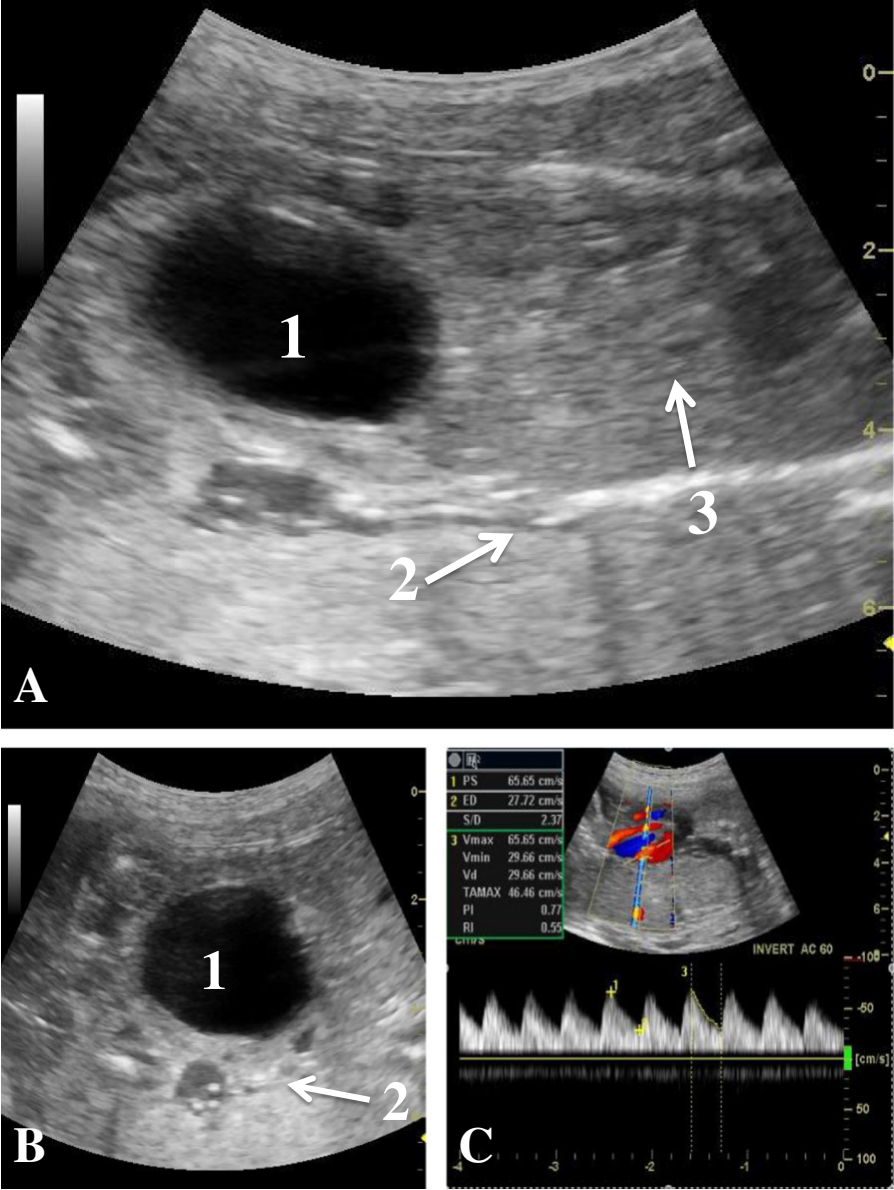
**Prenatal ultrasound findings of a case of prune-belly syndrome in Papua New Guinea. A**. Sagittal view – 1) megacystis, 2) anhydramnios, and 3) marked compression of abdominal and thoracic contents by the bladder. **B**. Coronal view - 1) megacystis and 2) anhydramnios. **C**. Umbilical artery Doppler at an estimated 22^+1^ weeks’ gestation.

All scans confirmed a single fetus with a normal fetal heart rate (152 – 158 beats per minute), a large and distended fetal bladder (megacystis), anhydramnios, and compressed fetal abdominal and thoracic contents (Figure 1). In view of the observed abnormalities only head circumference, biparietal diameter and femur length measurements were included on first scan to estimate gestational age (EGA): using standard charts [22], the EGA was 22^+1^, which was in agreement with gestational age by dates. During the same ultrasound examination the distended bladder measured 4.6 × 3.6 × 4.8 cms. The keyhole sign [9] was not observed, fetal sex could not be determined but there was evidence of concomitant ureteric dilatation (not shown). Umbilical artery flow indices were abnormal for gestational age on Doppler performed at second scan: the systolic:diastolic ratio (2.37), resistance index (0.55) and pulsatility index (0.77) were all below the 5^th^ percentile using standard reference charts [23-25]. These indicators of a low placental vascular resistance have not been confirmed as predictors of fetal outcome [26], and Doppler findings were not used in clinical decision making.

The mother was admitted to hospital at 24+^3^ weeks’ gestation for further assessment and a provisional diagnosis of megacystis and anhydramnios secondary to urethral obstruction or atresia was made. The mother (partner unavailable) was informed of the likely poor prognosis. She was counselled about strategies for further care, which included conservative management and termination of pregnancy using misoprostol, as well as palliative treatment versus resuscitation at delivery. The mother opted for non-interventional management and neonatal resuscitation if signs of life were present at birth. She was discharged and outpatient follow-up by both study and hospital clinicians was arranged.

The mother returned for one more antenatal visit at 26+^5^ weeks’ gestation during which she was provided with IPTp (SP as per national protocol), and iron and folic acid supplements and albendazole to treat her worsening anaemia (haemoglobin = 7.6 g/dL). Observations remained within normal range (35.8°C, blood pressure 98/51, heart rate 65, respiratory rate 20), and the fundal height was 25 cms.

**Figure 2:**
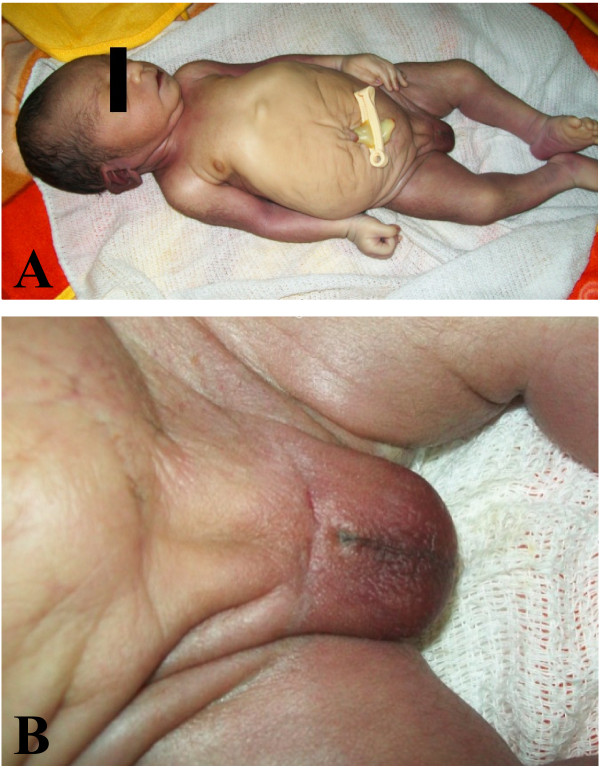
**Clinical findings. A**. Newborn’s general appearance – prune-belly syndrome characteristics include a distended wrinkled abdomen secondary to absent abdominal musculature, and a small chest secondary to lung hypoplasia. **B**. Abnormal male genitalia: undersized, underdeveloped scrotum with absent testes (cryptorchidism), penile aplasia and absent urethral meatus.

The mother presented to the Provincial General Hospital in spontaneous labour at 34 weeks’ gestation. On arrival fetal lie was longitudinal, presentation was cephalic and on baseline vaginal examination her cervix was 2cms dilated, fully effaced and membranes were intact. The fetal heart rate was 168 beats per minute. Maternal observations were normal (temperature 36.3°C, blood pressure 107/66, pulse 74, respiratory rate 20). Shortly after arrival she entered the active phase of first stage of labour and after five hours the cervix was fully dilated. The timing of rupture of membranes was not documented, and absence of liquor was noted at full dilatation. She delivered an infant weighing 2010 g (head circumference 290 mms, abdominal circumference 310 mms, length 440 mms) after 20 mins of second stage labour. At birth the patient had a pulse rate <100 beats per minute, an irregular respiratory rate of approximately 30 breaths per minute, and a poor response to stimulation (documented Apgar scores 3/1 and 5/5). Neonatal resuscitation was attempted by midwivery staff, oral suction was performed, and ventilatory support for the patient was provided using a bag-valve mask supplied with oxygen at a flow rate of 0.5 L/min*.* Despite these efforts the patient became increasingly apnoeic and cyanotic. Attending midwives were unable to pass a nasal oxygen catheter. A decision for conservative management was made after 20 minutes of resuscitation. The infant died two hours after delivery. On physical examination of the newborn performed by a research clinician multiple abnormalities were noted: the abdomen was distended, of prune-like appearance (wrinkled, lose skin) and anterior abdominal wall musculature was absent (Figure 2A); the male genitalia were severely abnormal, with notable absence of the urethral meatus, penile aplasia, and a small scrotum with cryptorchidism (Figure 2B); the chest was significantly small, consistent with lung hypoplasia secondary to anhydramnios (Figure 2A); and the nasal passages were either stenotic or atretic, although choanal atresia could not be confirmed. No other abnormalities were observed. Facilities for detailed postmortem examination were unavailable. Since the patient was taking part in a clinical trial assessing the safety of SP and azithromycin in second and third trimester of pregnancy, this case underwent an in-depth review by the trial’s safety team: in view of the diagnosis of PBS and its onset in early fetal development, as well as the short interval between drug administration and detection by ultrasound, a study drug-linked congenital abnormality was excluded. Clinical findings and images were discussed with an external consultant neonatologist (SR) who supported a diagnosis of PBS. The parents subsequently received further counseling from both hospital and study doctors, and the mother was discharged the following day.

## Conclusions

To our knowledge, this is the first case of PBS formally reported from PNG and other resource-limited regions in Oceania. In addition, it is the first report from PNG of prenatal diagnosis of a severe congenital abnormality using ultrasound technology.

The burden of birth defects in low-resource countries remains relatively under-researched and has been recognised as a target for studies and surveillance [27,28]. Information on the prevalence and characteristic of congenital abnormalities in PNG is limited [14-17]. Reporting and analysis of congenital abnormalities from low-resource environment such as PNG could be improved by establishing an international online passive surveillance system. Here, clinicians could submit case reports online and receive additional information and diagnostic support.

The ultrasound findings of anhydramnios and megacystis, in combination with the notable absence of a urethral meatus, a hypoplastic chest and the absence of anterior abdominal wall musculature on physical examination, strongly support a diagnosis of PBS in this patient, despite the lack of more detailed ultrasound or pathology data to corroborate these findings. Clinicians did not have the necessary training and experience to confidently detect PBS-related abnormalities on ultrasound, such as polycystic or dysplastic kidneys, hydronephrosis, lung hypoplasia, fetal ascites, and absent abdominal wall musculature [7,9], and autopsy facilities and a pathologist were unavailable.

PBS comprises a spectrum of mild to severe presentations [7]. This patient’s prenatal features of persistent mid-second trimester anhydramnios and megacystis pointed towards the uniformly lethal end of the spectrum of the syndrome [29,30]. Although the final diagnosis could only be made after delivery, a poor prognosis for the fetus was evident based on the observed ultrasound features alone. Furthermore, capacities for complex postpartum surgical interventions [8] are largely absent in PNG. In such clinical circumstances the survival of the fetus is very unlikely and he should be considered a dying patient [31]. Given its detection before fetal viability mothers ‘should be explained the nature and prognosis of the anomaly and offered both continuation of the pregnancy or induced abortion’ [31]. This was done in this case, and both mother and clinicians opted for non-intervention and monitoring. However, in order to reduce anxiety and the risks of over- or under-interventions, a more guarded response to parents may be more appropriate when significant uncertainties regarding the prognosis remain. Further advice should be sought when possible (e.g. ultrasound images could be sent elsewhere for verification). If a clear prognosis cannot be established, delivery should be awaited and mothers counseled accordingly. Intervention may then only become necessary should the fetal abnormality pose a threat to the mother’s health, such as hydrocephalus, increasing the risk of obstructed labour.

The limitations of ultrasound, and the limitations of the skill set of those performing ultrasound, must be highlighted to parents prior to scanning. In hospital practice, ultrasound complements, not replaces, routine clinical history and examination. Clarifying the purpose of the scan with the mother (e.g. dating the pregnancy) and reiterating that a scan does not rule out problems (congenital abnormality, obstetric complications) is very important in order to ensure that no harm is done. This is even more important when ultrasound is used as part of medical research. The mother of the patient was counseled to this effect at enrollment into the trial, was immediately withdrawn from the study upon detection of the abnormality, and was promptly referred to, and reviewed by, the most senior obstetric doctor in the region. The introduction of ultrasound in obstetric practice in PNG somewhat compares to introduction of a completely new medical technology to clinical practice in a high-income country [32]: it is the clinicians’ responsibility to be adequately trained and experienced prior to using information gathered through safe application of this technology to guide subsequent patient management. Ultrasound can assist doctors greatly but both mothers and doctors must recognise the limitations of the operators’ skill set and the technology overall. Introducing a national training curriculum for obstetric and gynaecological ultrasound could assist doctors in guiding and standardising their scanning practice.

It is likely that the large majority of congenital abnormalities detected prenatally in PNG will be managed conservatively. Induced abortion may rarely be opted for by parents because termination of pregnancy for socio-economic reasons is illegal in PNG and subject to severe legal punishment. The only circumstance where termination is legal is when undertaken by a specialist to save the life of a pregnant woman (includes preservation of both physical and mental health) [33]. In view of the current law (which is based on a British law from 1861 [33], now considerably amended in the UK), active management of this case (e.g. medical termination of pregnancy using misoprostol) could have been illegal. Future use of ultrasound technology for prenatal diagnostics in PNG should be carefully implemented in light of existing laws and ethical considerations.

Prenatal diagnosis of a severe congenital abnormality with poor prognosis for survival post-delivery must prompt clinicians to counsel parents regarding resuscitation of the newborn. Parents’ wishes must be respected, but counseling should explain the rationale behind no resuscitation or palliative treatment when there is sufficient certainty of a lethal outcome. In our case the mother’s request for neonatal resuscitation to be performed if signs of life were present at birth was respected. National guidance is not available but could be based on the principle that attempting neonatal resuscitation could be guided not only ‘*by aetiology of the baby’s condition, but should take into account the expected outcome, what is thought to be the best interests of the baby, and the wishes of parents*’ [34].

PNG is one of the most culturally diverse countries in the world. In addition to mapping the burden of birth defects in PNG, future research needs to evaluate the acceptability of ultrasound as part of antenatal care amongst women in PNG. Such findings can assist with developing culturally-appropriate guidelines, as observed elsewhere [35]. Similarly, understanding beliefs and attitudes towards birth defects, and the impact of the latter on the acceptability of ultrasound, clinical research, induced abortion and palliative treatment of affected fetuses is urgently required.

In summary, prune belly syndrome, a rare congenital malformation, occurs in Papua New Guinea. Monitoring and reporting of birth defects in PNG could be improved. Women undergoing antenatal ultrasound examinations must be carefully counseled on the purpose and the limitations of the scan. The increasing use of obstetric ultrasound in resource-limited settings such as PNG will inevitably result in a rise in prenatal detection of congenital abnormalities. This will need to be met with adequate training, referral mechanisms and better knowledge of women’s attitudes and beliefs on ultrasound and birth defects. National medico-legal guidance regarding induced abortion and resuscitation of a fetus with severe congenital abnormalities is required.

### Consent

Written informed consent for publication of this case report and accompanying images was obtained from the patient’s parents. A copy of the signed informed consent form is available for review by the Series Editor of BMC Pediatrics.

### Ethical approval

The mother of the infant gave informed written consent to participate in a prevention of malaria in pregnancy clinical trial (IPTp study), which was approved by the PNG IMR Institutional Review Board, PNG Medical Research Advisory Committee (MRAC) and the University of Melbourne Royal Melbourne Hospital Human Research Ethics Committee. The trial acts in compliance with the current revision of the Declaration of Helsinki, and with the International Conference for Harmonisation Good Clinical Practice (ICH-GCP) regulations and guidelines.

## Competing interests

The authors declare that there are no competing interests, either financial or non-financial, that could be perceived as prejudicing the impartiality of the research reported.

## Authors’ contributions

MO, JB, NNH, HWU and RW were involved in the clinical management of this patient and collected clinical details and photographs of this case report. MO, HWU, SR, RW, ML, JB and AJU reviewed the literature, and drafted the manuscript. All authors read and approved the final manuscript.

## Pre-publication history

The pre-publication history for this paper can be accessed here:

http://www.biomedcentral.com/1471-2431/13/70/prepub
